# Challenges of Surgical Treatment of Atypical Giant Choledochal Cyst with the Absence of Gallbladder in Adult Patient

**DOI:** 10.1155/2022/9605612

**Published:** 2022-04-20

**Authors:** Ismar Rasic, Nermin Mahmutovic, Samir Custovic, Nedim Hasic, Ali Gavrankapetanovic, Edin Beciragic, Sanela Brzika

**Affiliations:** General Hospital “Prim. Dr. Abdulah Nakas”, Department of Surgery, Sarajevo, Bosnia and Herzegovina

## Abstract

Choledochal cysts (CCs) are rare congenital anomalies in the form of cystic dilatation of any part of the biliary tree, and they rarely reach the size over 10 cm. We present a case of a 51-year-old female with a one-year history of abdominal pain and palpable mass in the epigastrium and right upper abdomen. Diagnosis of giant CC was made using magnetic resonance cholangiopancreatography. Intraoperatively, a large CC without gallbladder (no previous cholecystectomy was performed) was found under the liver pushing the surrounding structures, extending into the intrapancreatic portion of bile ducts and leading to the destruction of the backside of the pancreas head. Complete excision of CC and biliopancreatic reconstruction was achieved by Roux-en-Y pancreaticojejunostomy and hepaticojejunostomy. This case report describes an extremely rare association between an atypical giant choledochal cyst and gallbladder agenesis.

## 1. Introduction

Choledochal cysts (CCs) represent a malformation of the biliary tract in the form of an abnormal cystic dilatation of the biliary ducts. They can occur in any part of the biliary system, most often in the main portion of the common bile duct. The incidence of choledochal cysts from 1 : 100,000 to 1 : 150,000 classifies them as rare diseases. In most cases, they represent a surgical problem in childhood [1], but in 20% of cases, they can also be found in adults, with a higher incidence in females [2].

Todani classification from 1977 is the basic system of classification of CCs based on the distinction between five major types and several subtypes of CCs [3]. Choledochal cysts of type I (75-85%) involve dilatation of the common bile duct. Type II CC (2-3%) appears as an extrahepatic supraduodenal diverticulum. Type III CC (1.4-5%) is intraduodenal diverticulum, while cysts of type IV (10-19%) represent intrahepatic and extrahepatic dilatation of the bile ducts (type IVa) or multiple cysts limited to the extrahepatic bile duct (type IVb). Type V CC (Caroli disease) refers to multiple segmental intrahepatic cystic biliary dilatations. Surgery is the treatment of choice to avoid the risk of complications such as pancreatitis, choledocholithiasis, or cholangiocarcinoma and includes complete cystic excision (including gallbladder) with biliary-enteric reconstruction.

In adults, choledochal cysts are rarely symptomatic, but there is the risk of malignant transformation of the biliary tree. Although variations in the size of bile cysts have been shown, several cases of giant cysts over 10 cm have been reported in the literature [4–6].

This case report describes an extremely rare association between gallbladder agenesis and atypical giant CC and the complexity of its surgical treatment.

## 2. Case Presentation

A 51-year-old woman was admitted to our hospital for surgical treatment of a large CC. She had no previous abdominal surgery. For the past year, she had suffered from pain in the right side of the abdomen with an extension to her back. Four months before admission, the patient had first jaundice attack and underwent endoscopic retrograde cholangiopancreatography (ERCP) with biliary stent implantation.

**Figure 1:**
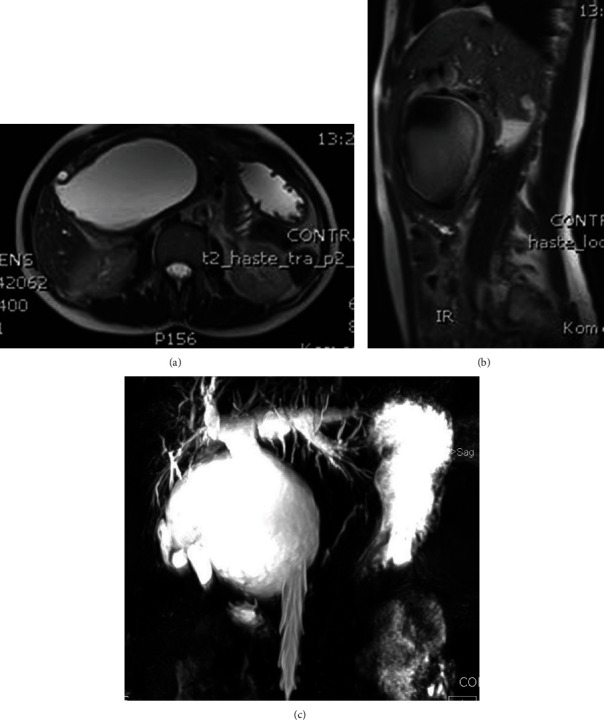
Magnetic resonance cholangiopancreatography finding. MRCP displays a large choledochal cyst in axial (a) and coronal (b) view of the abdomen. Cyst of the common bile duct and common hepatic duct, with partial dilatation of left and right hepatic ducts, without existing gallbladder (c).

Upon admission to our hospital, the patient was anicteric, without fever. A large abdominal mass was palpated in the epigastric region and right upper quadrant of the abdomen. Laboratory test showed serum C reactive protein (CRP) values of 85 mg/L (ref. range: 0-5 mg/L), gamma glutamyl-transferase (GGT) level of 220 U/L (ref. range: 5-85 U/L), and slightly elevated aspartate aminotransferase (AST) level of 55 U/L (ref. range: 15-37 U/L), whereas other parameters were within normal range. A magnetic resonance cholangiopancreatography (MRCP) revealed a large cystic formation of extrahepatic bile ducts with a maximum diameter of 141 mm, without a visible gallbladder, and with partial dilatation of left and right hepatic ducts (Figure 1).

**Figure 2:**
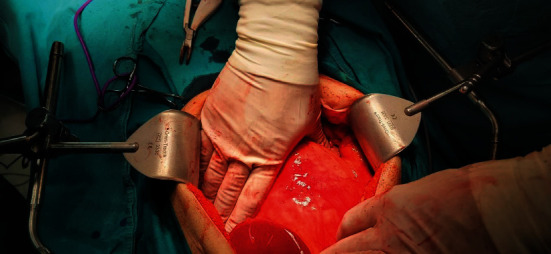
Intraoperative finding of a large choledochal cyst.

Intraoperatively, a large fibrous cystic formation (Figure 2) was found located underneath the liver with absence of the gallbladder.

**Figure 3:**
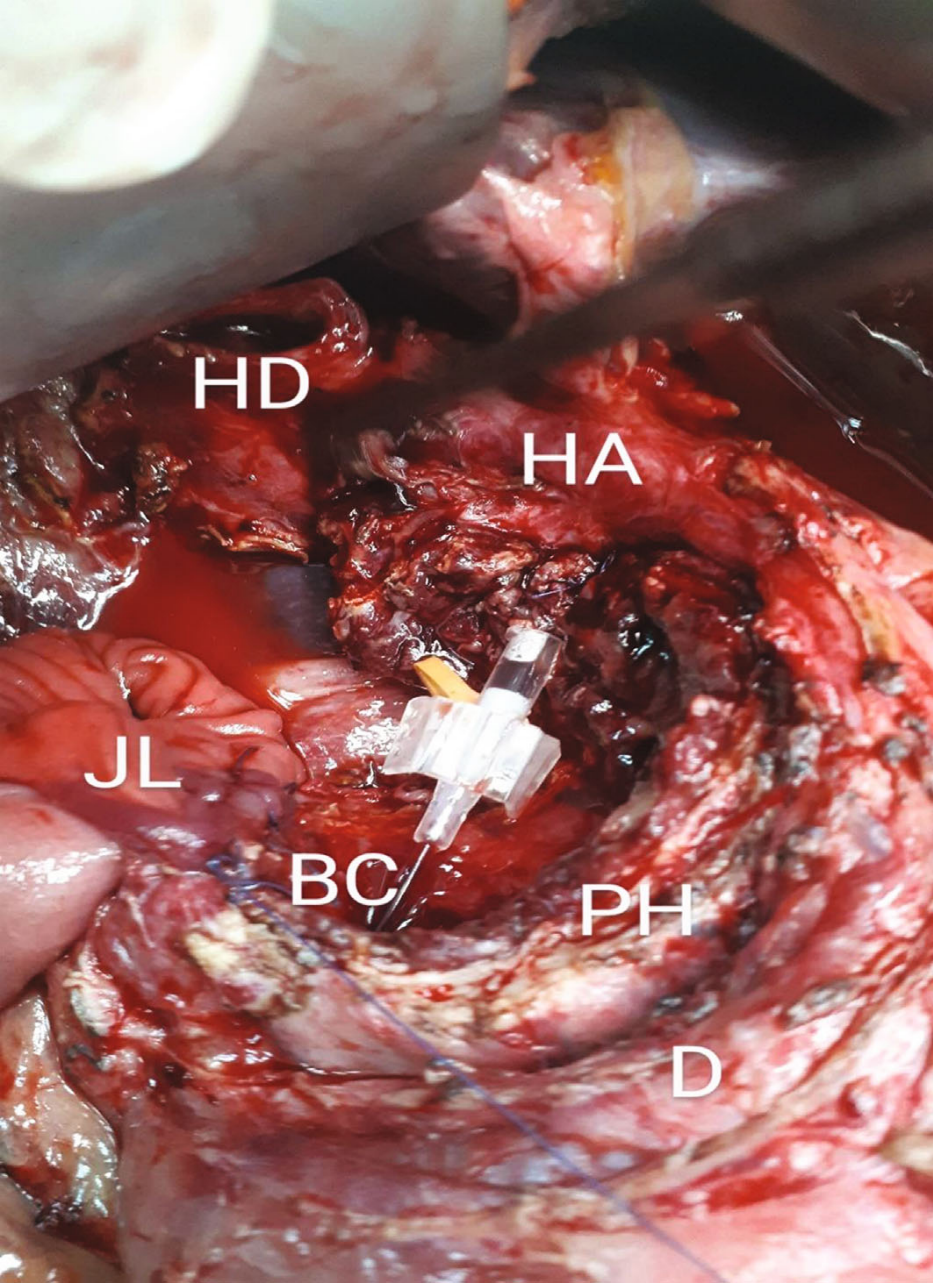
Operating field after cyst resection. Destruction of the posterior side of the pancreatic head with suppressed ventral duodenum. Common bile duct cut immediately below the hepatic ducts confluence. Biliary cannula temporarily placed in the ampulla of Vateri for intraoperative orientation (HD: hepatic ducts confluence; HA: hepatic artery; JL: jejunal loop; PH: pancreatic head; D: duodenal frame; BC: biliary cannula).

**Figure 4:**
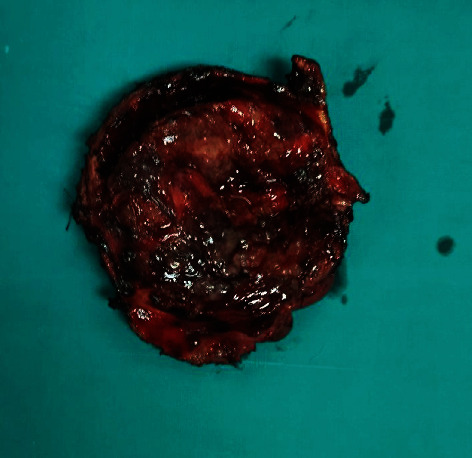
Specimen of completely excised choledochal cyst.

The cyst was found to push the duodenum from the native position forward, extend into the intrapancreatic portion of the bile duct, and lead to the destruction of the posterior side of the pancreatic head. The intrapancreatic cystic mass extended through the pancreas to 1.5 cm from the ampulla Vateri. It was separated from the pancreas by resection in healthy pancreatic tissue. The cyst was found to have grown into the hepatoduodenal ligament and was carefully extirpated from it. The common bile duct was cut above the cyst and just below the confluence of the hepatic ducts (Figure 3). The purulent contents that filled the entire choledochal cyst were evacuated from the bile ducts. After complete excision of the cyst and its macroscopic examination, it was sent for histological analysis (Figure 4).

**Figure 5:**
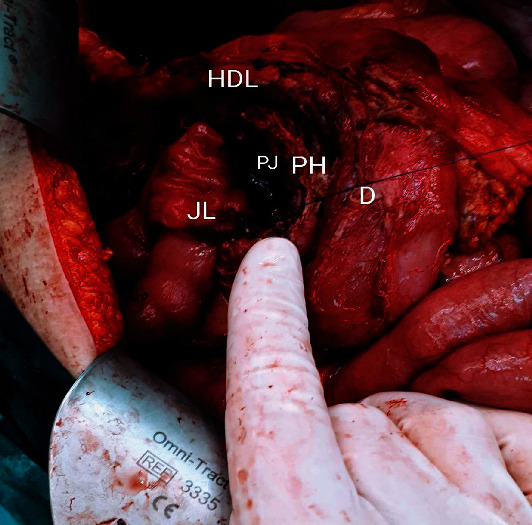
Intraoperative view of the performed Roux-en-Y pancreaticojejunostomy (on the pancreatic head defect) (JL: jejunal loop; PH: pancreatic head; D: duodenum; PJ: pancreaticojejunostomy; HDL: hepatoduodenal ligament).

In the next step, the retrocolic Roux-en-Y pancreaticojejunostomy on the pancreatic head defect was performed (Figure 5), followed by hepaticojejunostomy.

Immediately after the operative act, the patient was extubated. The patient was monitored after surgery for possible postoperative complications. She was hemodynamically stable, without breathing and lung complications. There were no complications in terms of bile leakage, pancreatic fistula, intra-abdominal infection and abscess or delayed wound healing. The patient was discharged on the fourteenth day following surgery. Control laboratory tests showed the following: CRP 28 g/L, GGT 118 U/L, b in addition to normal other laboratory findings.

Histopathological examination revealed markedly fibrous thickened cyst wall with signs of chronic inflammation. Immunohistochemical analysis ruled out the existence of malignant alteration.

## 3. Discussion

Classic triad symptoms of CC include abdominal pain, jaundice, and palpable abdominal mass. Adult patient with CC are mostly asymptomatic or with abdominal pain in right upper quadrant. Our patient became symptomatic only at the age of fifty, with abdominal pain and first jaundice attack, when CC was diagnosed.

Todani classification of CCs into five main types and several subtypes is widely used as a guide for surgical treatment of CCs. Still, associated hepatobiliary pathology can be suddenly detected during the surgical treatment itself and affect its course.

The described CC is characterized not only by its enormous size (141 mm) but also by anatomical changes in the form of a large dilatation of the extrahepatic biliary tree with the absence of the gallbladder. In addition, this cyst is characterized by the extension along the intrapancreatic portion of the bile duct and the complete destruction of the posterior side of the pancreatic head. So, it cannot be classified in any classification group of choledochal cysts, according to Todani. This CC has in part the characteristics of type IVa CC but differs from it by the congenital absence of the gallbladder and with its intrapancreatic extension.

The resection of the cyst was very demanding due to its size, pronounced wall thickness, and intimate contact with surrounding anatomical structures. After the cyst resection, in the existing defect of the pancreatic head, involvement of the pancreatic duct could not be ruled out, so an atypical pancreaticojejunostomy was performed, followed by a biliary-enteric diversion on the same jejunal loop.

Choledochal cysts are rare congenital abnormalities of the bile ducts. Additionally, isolated gallbladder agenesis is also a rare anatomical variation, with an incidence of 10-65 per 100,000 and three times more common in women. Many adults with gallbladder agenesis remain asymptomatic for life, and diagnosis of this biliary anomaly is usually established incidentally during investigation or surgery for another disease.

The combination of these two anomalies is extremely rare, described in only a few cases in adults [7–12] in which CCs type I or type IV were associated with gallbladder agenesis. However, to our knowledge, a case of congenital absence of the gallbladder associated with an atypical giant CC that led to complete destruction of the posterior side of the pancreatic head, and symptomatically manifested only in the fifth decade of life, has not been previously reported.

## Data Availability

The data used to support the findings of this study are included within the article.
